# Reconceptualize tall-cell variant papillary thyroid microcarcinoma: From a “sonographic histology” perspective

**DOI:** 10.3389/fendo.2022.1001477

**Published:** 2022-11-08

**Authors:** Yongyue Zhang, Fang Mei, Xiaoxi He, Jing Ma, Shumin Wang

**Affiliations:** ^1^ Department of Ultrasound, Peking University Third Hospital, Beijing, China; ^2^ Department of Pathology, Peking University Third Hospital, Beijing, China

**Keywords:** sonographic histology, tall cell variant, papillary thyroid microcarcinoma, aggressive subtype, Thyroid Imaging Reporting and Data System (TI-RADS)

## Abstract

**Objective:**

This study aimed to examine the relationship between sonographic features and histological manifestations in the tall-cell variant of papillary thyroid microcarcinoma (TCV-PTMC), thus proposing the concept of “sonographic histology” and examine its value in the clinical management of the aggressive tall-cell variant.

**Methods:**

This study retrospectively included 104 participants who were admitted to Peking University Third Hospital from 2015 to 2022 and were histopathologically confirmed as having TCV-PTMC or classical PTMC. We mainly compared the general characteristics, sonographic characteristics, and pathological specimens between the two cohorts.

**Results:**

Hypoechoic nodules with a localized central isoechoic lesion and hypoechoic halo around nodules were most often observed in TCV-PTMC, which correlated with circumferentially distributed tumor epithelium and densely distributed tumor stroma histopathologically. Additionally, TCV-PTMC showed nodules with a more regular margin and less microcalcification than classical PTMC, which led to an underestimation of the risk of TCV-PTMC.

**Conclusion:**

The good association between the ultrasound echo pattern and tissue cell arrangement was defined as sonographic histology in this study and can be applied in the preoperative identification of TCV-PTMC. This concept may provide novel insight for the identification of special subtypes of thyroid tumors and may modify pitfalls of the Thyroid Imaging Reporting and Data System in aggressive variants of microcarcinoma.

## Introduction

The worldwide incidence of thyroid cancer has continuously increased in the last few decades, but the mortality rate has remained flat. The most prominent reasons for this lack of a decrease in mortality are the widespread adoption of ultrasonography (US) (1) and the development of high-frequency transducers, which enable the imaging of more papillary thyroid microcarcinomas (PTMCs) (2). Notably, except for the main two-dimensional parameters included in the Thyroid Imaging Reporting and Data System (TI-RADS), the use of the highest frequency that has adequate tissue penetration can clearly visualize part of the microstructure. Many sonographers have attempted to interpret the local features inside lesions of the thyroid from a pathological histological perspective and have established some excellent agreement between sonographic features and histological manifestations. These features include a hypoechoic halo as shown by sonography versus a fibrous capsule in follicular tumors as shown by histology (3), microcalcifications versus psammoma bodies (4), and extensive amyloid deposition versus anechoic areas in medullary thyroid carcinoma nodules (5). We define the excellent correspondence between ultrasound echo patterns and tissue cell arrangement as “sonographic histology” in this study. Despite being proposed for the first time, numerous studies linking specific US signs to pathology have demonstrated the importance of this concept for the risk assessment of thyroid nodules (6–8).

Papillary thyroid carcinoma (PTC) that is ≤1.0 cm in the largest dimension is defined as PTMC in the World Health Organization (WHO) classification (9). Although prospective clinical trials of active surveillance for low-risk PTMC have reported favorable outcomes, a recent consensus still recommended that immediate surgery should be conducted when the high-risk subtypes are suspected on cytology owing to their aggressive behavior (10). As a typical representative of a high-risk variant of PTC, the tall-cell variant (TCV) is more aggressive than classical PTC (11) because of its larger tumor size, higher frequency of extrathyroid extension (ETE), and poorer survival (12). TCV was described by the latest WHO guidelines (2017) as having a composition of >30% of cells that are two to three times as high as they are wide (13). According to the current guidelines (10), high-risk TCV nodules are suitable for early surgery rather than an active surveillance strategy even at the stage of microcarcinoma. However, there are difficulties in the preoperative diagnosis of this subtype. To date, only a few studies have reported distinctive sonographic features for this variant, but most of these overlap with US findings in classical PTC (14). In the concept of sonographic histology mentioned above, a higher surface resolution could provide new opportunities. Previous studies have reported that the relative proportions of tumor stroma and tumor epithelium are related to tumor cell survival, growth, and metastatic spread (15). Therefore, these histological features might be potential diagnostic biomarkers of aggressive subtypes. By comparing US with pathological slides, sonographers may achieve a deeper understanding of growth patterns and behavioral processes of specific sonographic representations rather than solely focusing on the limited morphological parameters of the TI-RADS.

In this study, we retrospectively reviewed the demographic, histopathological, and sonographic characteristics of a series of patients with TCV-PTMC and classical PTMC (C-PTMC). First, we investigated how US manifestations are associated with histological characteristics, which may indicate the biological behaviors of these TCV-PTMCs. Second, we briefly discuss the diagnostic value of sonographic histology in the current management of aggressive subtypes of PTMC.

## Materials and methods

### Selection of patients

**Figure 1:**
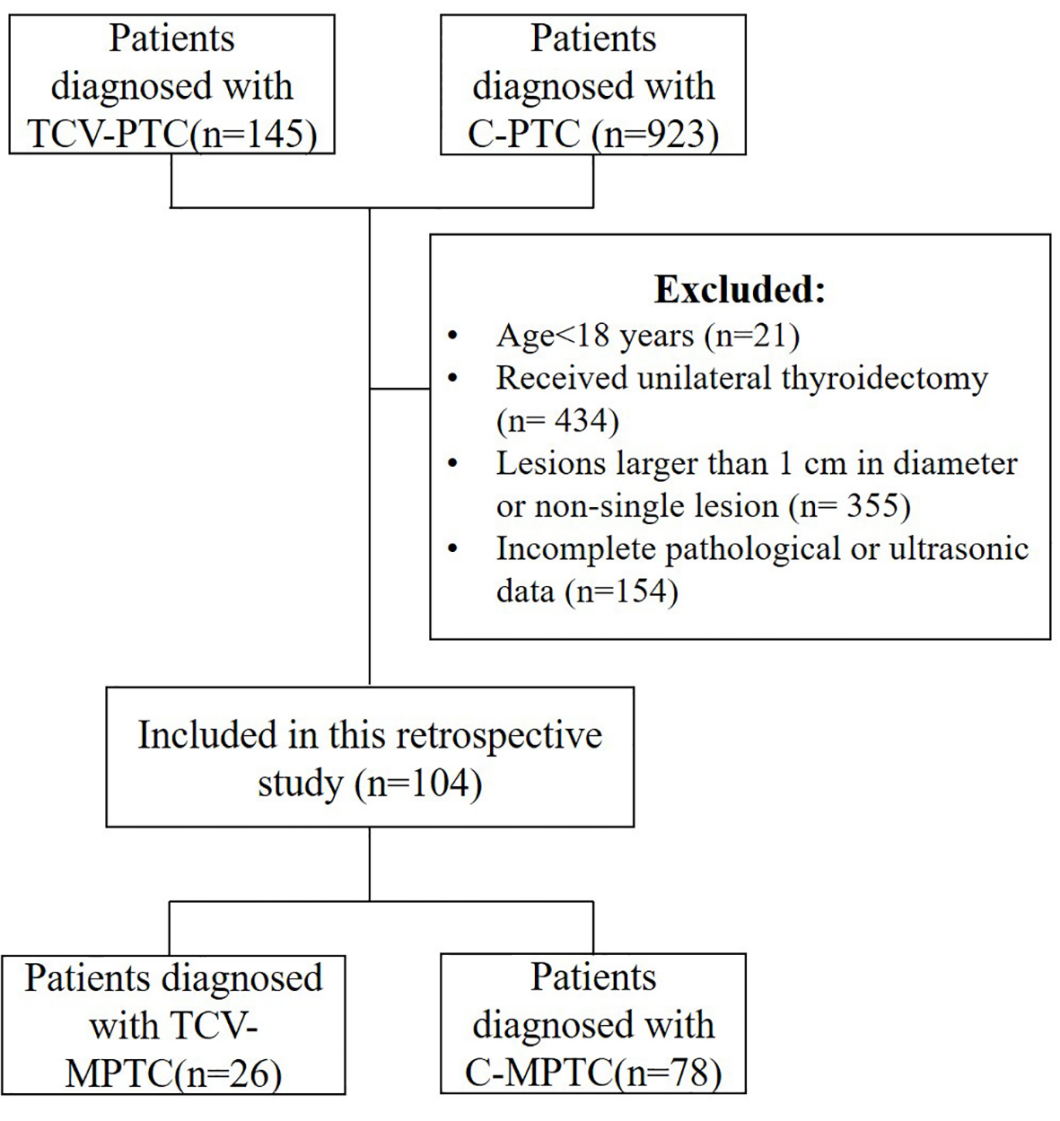
Flow chart of the enrolled patients.

The Institutional Review Board approved this retrospective study, and the need for informed consent was waived. The analyzed data were retrieved from the medical records. We included 145 patients with TCV and 923 with classical PTC who were treated at our hospital between January 2015 and March 2022. The inclusion criteria were rigorous, with eligibility limited to patients with surgical total thyroidectomy. The patients were evaluated by US before thyroidectomy and were found to have a single nodule that was histopathologically confirmed as TCV-PTMC or C-PTMC. The final histopathology report was the gold standard for inclusion in the analyses. The histopathological criteria for the diagnosis of TCV-PTC were according to the latest WHO guidelines as follows: a tumor composed of >30% tall cells, a tall-cell height at least twice its breadth, an eosinophilic tall-cell cytoplasm, and nuclear features characteristic of PTC. Representative pathological slides were independently reviewed by two pathologists. Finally, 104 patients (26 with TCV-PTMC and 78 with C-PTMC) were selected for the analysis (**Figure 1**). Clinical and histopathological data included in the analyses were age, sex, body mass index (BMI), the percentage of the stroma (PS) in the tumor and the percentage of epithelial tissue (PE) in the tumor, the presence or absence of Hashimoto’s thyroiditis (HT), lymph node metastasis, and ETE. Among these, ETE was defined as a primary tumor extending through the thyroid capsule to perithyroidal soft tissue (16). The histopathological features of PE and PS were classified by experienced pathologists as follows: level 1, the stromal ratio or epithelial cell ratio occupied 0%–33.3% of the nodule; level 2, 33.4%–66.7%, and level 3, 66.8%–100%.

### US evaluation

The preoperative US was performed using a 5–12-MHz linear array probe (iU22; Philips Medical Systems, Amsterdam, The Netherlands). All US images were evaluated by two radiologists who were blinded to the histological results. The scanning protocol in all patients included longitudinal and transverse real-time imaging of the thyroid nodules. The nodule size and suspicious US features, such as echogenicity, the margin, the presence or absence of microcalcifications, and the length/width ratio of nodules were recorded. The tumor size was measured along the maximum diameter in any sonographic view. The nodular margin was classified as regular or irregular. The height/width ratio of thyroid nodules was recorded as >1 or <1. The calcification was assessed with respect to its size and was classified as microcalcification (when there were tiny, punctate echogenic foci of ≤1 mm with or without posterior shadowing), which was classified as present or absent. Tumor echogenicity of the solid portion was assessed according to the normal thyroid parenchyma and strap muscles and classified as hyperechoic, isoechoic, or hypoechoic. We found local isoechogenicity in some hypoechoic nodules. The presence or absence of an isoechoic pattern was noted. Additionally, nodule subtypes were defined by the presence of central or noncentral isoechogenicity. The US findings were evaluated by the TI-RADS Committee of the American College of Radiology (17).

### Statistical analysis

Statistical analysis was performed using IBM SPSS (SPSS, version 24.0 for Windows; IBM Corp., Armonk, NY, USA). The TCV-PTMC and C-PTMC cohorts were compared using the chi-square test for categorical variables (sex, HT, PS, PE, ETE, lymph node metastasis, the margin, microcalcification, echogenicity, and the length/width ratio), and the two-sample *t*-test and the Mann–Whitney *U* test were used for continuous variables (age, BMI, size, and TI-RADS scores). A significant difference was defined as a *p*-value <0.05.

## Results

### Clinical and histopathological findings

After screening, the final population was composed of 77 women (TCV: 17, C-PTMC: 60) and 27 men (TCV: 9, C-PTMC: 18), with an average age of 43.1 years (TCV: 55.7, C-PTMC: 39.3). The clinical and histopathological results are shown in **Table 1**. The tumor epithelial ratio was higher and the tumor stromal ratio was lower in the TCV-PTMC cohort than in the C-PTMC cohort (both *p* < 0.05). Additionally, the patients with TCV-PTMC had an older age, a higher BMI, and a higher ETE ratio than those with C-PTMC (*p* < 0.05). There were no significant differences in sex, HT, or lymph node metastasis between the two cohorts.

**Table 1:** Clinicopathological characteristics of TCV-PTMC and C-PTMC nodules.

Feature	TCV-PTMC	C-PTMC	*t/Z/χ2*	*p*
	*N* = 26	*N* = 78		
Age (year)	55.73 ± 12.18	39.27 ± 12.14	5.98	0.000
BMI (kg/m^2^)	25.54 (24.11–28.18)	24.41 (21.78–26.57)	2.15	0.032
Gender			1.35	0.245
Male	9 (34.6%)	18 (23.1%)		
Female	17 (65.4%)	60 (76.9%)		
HT			3.36	0.067
Present	11 (42.3%)	49 (62.8%)		
Absent	15 (57.7%)	29 (37.2%)		
PS			9.02	0.011
Level 1	19 (73.1%)	31 (39.7%)		
Level 2	6 (23.1%)	34(43.6%)		
Level 3	1 (3.8%)	13 (16.7%)		
PE			9.02	0.011
Level 1	1 (3.8%)	13 (16.7%)		
Level 2	6 (23.1%)	34 (43.6%)		
Level 3	19 (73.1%)	31 (39.7%)		
ETE			9.344	0.002
Present	16 (61.5%)	22 (28.2%)		
Absent	10 (38.5%)	56 (71.8%)		
LNM			0.21	0.647
Present	16 (61.5%)	44 (56.4%)		
Absent	10 (38.5%)	34 (43.6%)		

TCV, tall-cell variant; PTMC, papillary thyroid microcarcinoma; BMI, body mass index; HT, Hashimoto’s thyroiditis; PS, percentage of the stroma; PE, percentage of epithelial tissue; ETE, extrathyroidal extension; LNM, lymph node metastasis.

**Figure 2:**
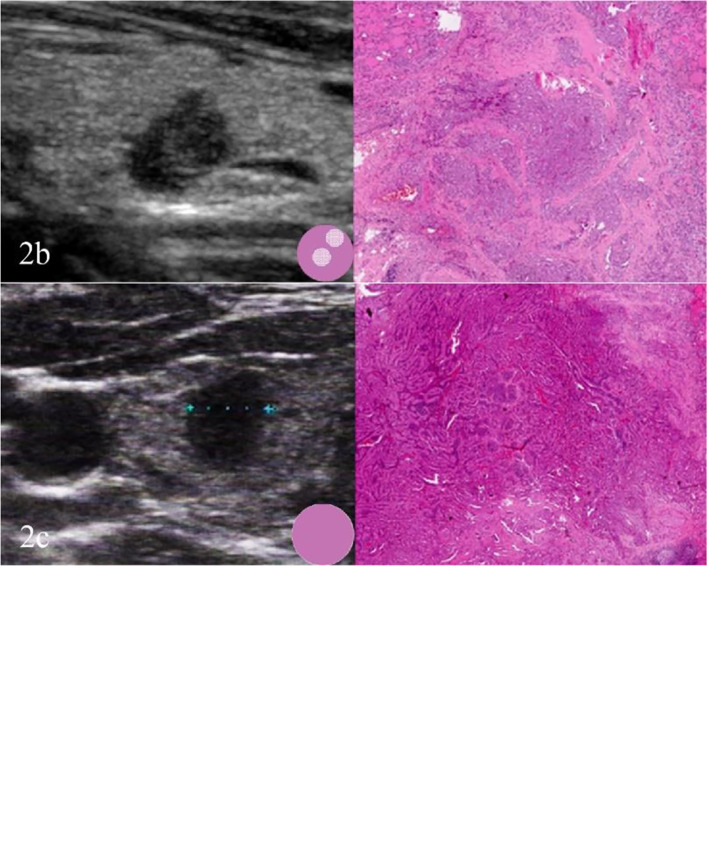
Three cases of TCV-PTMC. **(A)** US of the left thyroid gland in a 57-year-old woman shows a hypoechoic nodule with a localized central isoechoic region and a hypoechoic halo. This nodule has a regular margin and a diameter of 7 mm (left). Hematoxylin and eosin staining (×40) shows an irregular pink fibrous stromal center that is surrounded by epithelium (right). **(B)** US of the left thyroid gland in a 63-year-old woman shows a hypoechoic nodule with a localized noncentral isoechoic region. This nodule has an irregular margin and a diameter of 8 mm (left). Hematoxylin and eosin staining (×40) shows an irregular pink region indicating a randomly distributed stroma (right). **(C)** US of the right thyroid gland in a 44-year-old woman shows a hypoechoic nodule without a localized isoechoic region. This nodule has a regular margin and a diameter of 7 mm (left). Hematoxylin and eosin staining (×40): shows a hypercellular tumor with scant stromal components (right).

**Figure 3:**
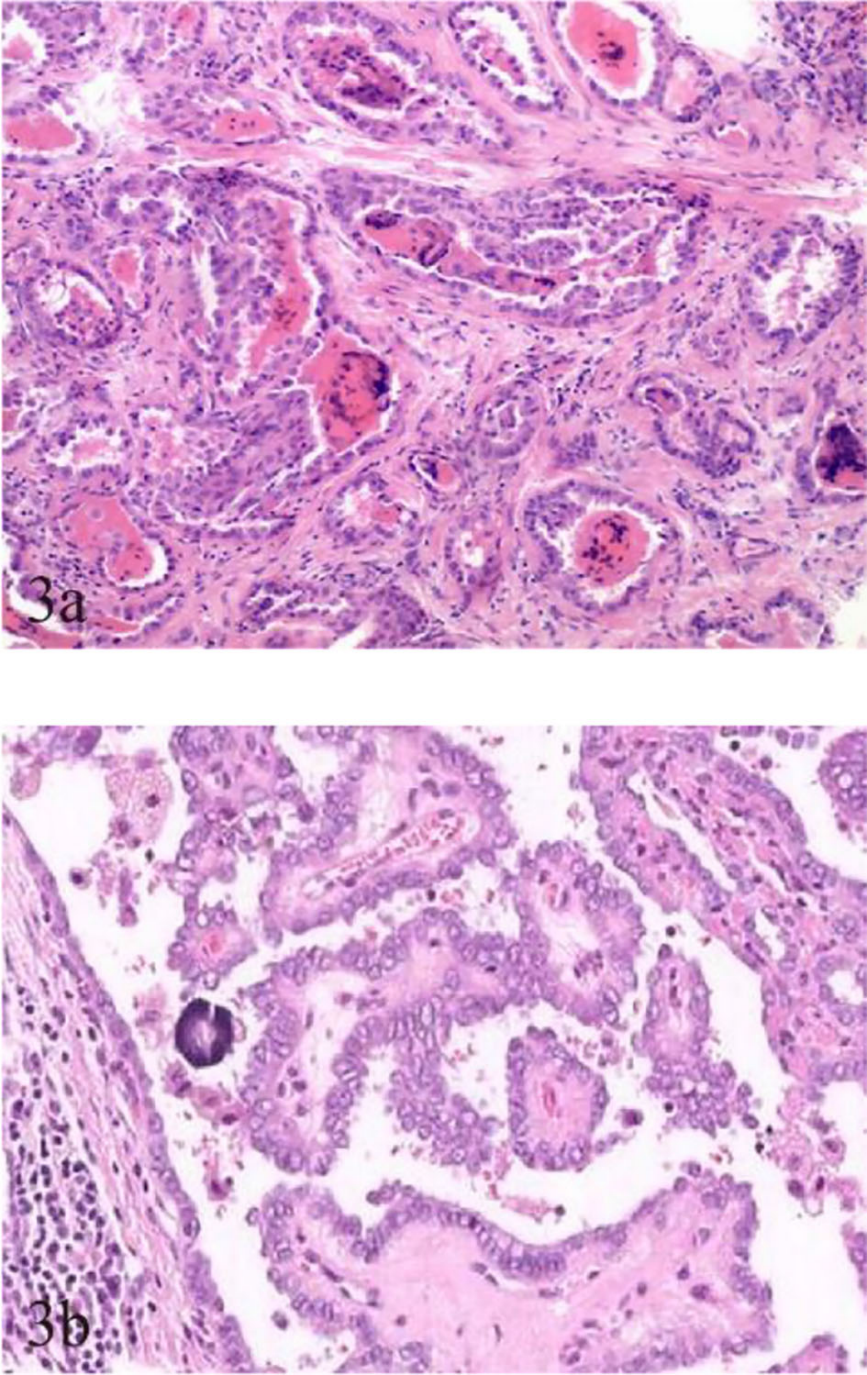
Pathological characteristics of TCV-PTMC and C-PTMC. **(A)** TCV in a 63-year-old woman with an unevenly distributed increased number of cellular fibrocytes and delicate collagenous areas (hematoxylin and eosin staining, ×200). **(B)** C-PTMC in a 48-year-old man with few evenly distributed fibrocytes and a prominent collagenized stroma (hematoxylin and eosin staining, ×400).

Fewer but more immature stroma with aggregate fibrosis and delicate collagen fibers were observed more frequently in TCV-PTMC than in C-PTMC; more tumor epithelial cells were always tightly clustered around the periphery of TCV-PTMC nodules, whereas immature tumor stroma was distributed in the central or noncentral region, or with a scanty stromal component in nodules (**Figure 2**). Unlike TCV-PTMC tumors with a high percentage of epithelial tissue and a low percentage of stroma, C-PTMC showed a distinct papillary structure and fewer epithelial tissues with a more fibrovascular core containing prominent and collagenized stroma, which appeared more evenly distributed and not tightly clustered (**Figure 3**).

### US findings

In this study, tumor nodules were defined by five parameters according to the TI-RADS, including echogenicity, composition, calcification, margin, and height/width ratio. A significant difference was found in the scores of TI-RADS between the two cohorts (*p* < 0.05). All of the tumor nodules included in the analysis were hypoechoic with or without local isoechogenicity. Patients with TCV-PTMC showed a greater presence of isoechogenicity (*p* = 0.015), and the isoechogenicity tended to be located in the central area (16 nodules, 76.2%) than in the noncentral area (five nodules, 23.8%) (*p* = 0.001) compared with those with C-PTMC (**Figures 2A, B**). Only five of 26 TCV nodules were hypoechoic without the presence of any isoechogenicity (**Figure 2C**).

Except for echogenicity, the margin of nodules in patients with TCV-PTMC showed a greater tendency to be well defined (*p* = 0.033) and had fewer microcalcifications (*p* < 0.01) than those with C-PTMC. There were 53 (67.9%) nodules with microcalcifications in patients with C-PTMC, but in those with TCV-PTMC, there are only nine (34.6%) nodules. The nodule size and the length/width ratio were not significantly different between the two cohorts (**Table 2**).

**Table 2:** Ultrasonic characteristics of TCV-PTMC and C-PTMC nodules.

Feature	TCV-PTMC	C-PTMC	*t/Z/χ^2^*	*p*
	*N* = 26	*N* = 78		
TI-RADS	6.80 ± 1.91	10.22 ± 2.42	10.19	0.001
Size (cm)	0.75 ± 0.20	0.71 ± 0.18	0.95	0.347
Margin			4.52	0.033
Regular	6 (23.1%)	6 (7.7%)		
Irregular	20 (76.9%)	72 (92.3%)		
Microcalcification			8.99	0.003
Present	9 (34.6%)	53 (67.9%)		
Absent	17 (65.4%)	25 (32.1%)		
Isoechogenicity			5.92	0.015
Present	21 (80.8%)	42 (53.8%)		
Absent	5 (19.2%)	36 (46.2%)		
Location of isoecho			11.53	0.001
Central	16 (76.2%)	13 (31.0%)		
Noncentral	5 (23.8%)	29 (69.0%)		
Ratio of length/width			1.85	0.173
>1	21 (80.8%)	52 (66.7%)		
<1	5 (19.2%)	26 (33.3%)		

TCV, tall-cell variant; PTMC, papillary thyroid microcarcinoma; TI-RADS, Thyroid Imaging Reporting, and Data System.

## Discussion

The appropriate extent of fine-needle aspiration or surgical resection for newly diagnosed PTMC remains contentious (18). Current guidelines recommend nonoperative approaches with active surveillance if the tumor is ≤1 cm in its greatest dimension and does not have any identifiable aggressive features (19). However, patients with aggressive variants should be treated with thyroidectomy instead of surveillance, regardless of the tumor size (20). The TCV is widely reported to be a typical example of an aggressive subtype of PTC with more ETE and distant metastasis and a poorer prognosis, even at the microcarcinoma stage (21, 22). Therefore, identifying TCV nodules preoperatively is crucial and deserves our attention. Although US is the preferred tool of choice for identifying PTMC, some research has suggested that most US characteristics of the TCV overlap with those in classical PTC (23, 24). Therefore, preoperative diagnosis of the TCV poses a challenge to sonographers. In our center, a few factors, such as the patient’s anxiety, a high-risk growth pattern, and poor follow-up, resulted in >50% of patients being diagnosed with PTMC and undergoing thyroidectomy. Therefore, we focused on the different subtypes of PTMC in this study and aimed to examine the unique features for the early identification of TCV-PTMC and the value of sonographic histology in thyroid US.

To date, most studies on TCV primarily focused on histological characteristics and the proportion of tall cells (25), whereas few studies examined the association between the sonographic findings of TCV and their histopathologic characteristics. In our series, TCVs manifested as unevenly distributed isoechogenicity and showed peripheral hypoechoic halos inside the nodules. These features showed a good correspondence with the pathological characteristics, such as concentration of the fibrous stroma in the central or noncentral zone and enrichment of peripheral epithelial cells (**Figure 2**). We defined this relationship as sonographic histology, which has not been reported elsewhere in the literature. The main physical basis of sonographic histology is differences in acoustic impedance between different tissues and morphologically variable alignment patterns. Sonographically, US waves lack reflective interfaces and have good acoustic transmission after passing through the abundant tumor epithelium, whereas more interfaces in the stromal component contribute to the formation of isoechogenicity. Previous reports have suggested that the amount of fibrous stroma in PTMC nodules determines whether the nodules are heterogeneous or homogeneous in terms of echogenicity (6). However, our findings suggest that the generation of heterogeneity is not only related to the amount of stroma but also to its arrangement. We observed the distinct isoechoic regions were exhibited in TCV nodules with low-proportion but locally aggregated stroma; in contrast, the presence of more but evenly distributed stroma made the isoechogenicity less obvious in C-PTMC nodules (**Figure 3**).

In our study, we identified three types of distribution of epithelium and the stroma in TCV-PTMC nodules. Among these, a central and dense distribution of the tumor stroma was the most commonly detected, with a highly cellular epithelium surrounding the periphery of nodules, and a “thick halo” was observed by US (**Figure 2A**). However, we found that the “halo” was an ambiguous concept, which was not considered for inclusion in the TI-RADS but has a unique value in histology (26). A halo may represent a fibrous pseudo-capsule in follicular tumors (27), an inflammatory reactive zone of hyperplastic nodules, or a dense epithelial cell population, as shown in our study. Tall cells located around the nodules increase infiltration into the surrounding tissue and the aggressiveness of the tumor. Therefore, we believe that a thick hypoechoic halo around suspected PTMC nodules should attract the suspicion of aggressive subtypes such as the TCV.

Our study showed that a well-defined margin observed by US was associated with a regular histological margin in the TCV-PTMC cohort. This finding resulted from homogeneous cell cycles and a rapid growth pattern of tall cells at the periphery of nodules. Furthermore, microcalcification, which may reflect psammoma bodies, had a lower incidence in patients with TCV-PTMC than in those with C-PTMC in our study. Psammoma bodies may lead to the degeneration or death of tumor cells and retardation of neoplasm growth (28, 29). Because of less stroma and a lower degree of hyalinization, the main process of calcification based on degenerative collagenized stroma may be largely blocked in TCV-PTMCs. Therefore, the mean score of the TI-RADS was considerably lower in patients with TCV-PTMC than in those with C-PTMC, which may be associated with a more regular margin and less microcalcifications. Importantly, lower TI-RADS scores may not attract sufficient attention from clinicians, and the severity of TCV may be underestimated. Therefore, we suggest that increasing attention to the internal microstructure inside nodules may be important for identifying TCV-PTMC or other aggressive variants.

With regard to the clinical management of suspicious TCV nodules, although most PTMCs are thought to be clinically indolent, our finding that TCV-PTMC exhibits a more aggressive behavior pattern, but shows lower TI-RADS scores than C-PTMC, is important. Researchers have proposed that PTMCs with different biological behaviors should be treated differently (30). We support the shortening of follow-up periods and more aggressively monitoring the growth of suspicious nodules. Further prognostic research on these suggestions is required in the future. Furthermore, we acknowledge that there are pitfalls regarding solely relying on the diameter of the nodule or TI-RADS scores to decide whether surgical removal should be conducted, which was supported by the findings of Kazaure et al. (20). Specific sonographic features, such as an abnormally thickened halo, have important diagnostic value for TCV-PTMC. With the wide availability of high-frequency US probes, sonographic histology can enable the early identification of high-risk PTMC subtypes and personalized management.

We also observed the interesting finding that patients with TCV-PTMC tended to have a higher BMI, and 17 of the 26 patients suffered from obesity simultaneously in accordance with the criteria published by the WHO in our study (31). To the best of our knowledge, the relationship between obesity and the occurrence of TCVs has not been previously reported, despite the convincing evidence that an excess BMI is associated with an increased risk of thyroid cancer (32). With the global prevalence of obesity, we believe that further research into the role of obesity and associated metabolic syndrome in aggressive variant subtypes of PTC is warranted in the future.

This study has some limitations. First, the study sample size was limited by the rigorous inclusion criteria. To meet the purpose of our study for the early diagnosis of microcarcinoma, only those with a single small nodule of <1 cm in size were included. Second, the US features of PTMC were only associated with histopathology and not with the patients’ outcomes. Further studies with a longer follow-up may provide more information about TCV-PTMC. In the future, more precise sonographic and pathological correspondence and appropriate preoperative risk stratification should be examined in patients with PTMC in prospectively controlled studies.

## Conclusion

In conclusion, the sonographic features of TCV-PTMC may reflect the histopathological findings to a certain extent and vice versa. The concept of “sonographic histology” indicates that the visualization of microstructure can reconceptualize thyroid nodules from a pathological perspective rather than relying only on the traditional TI-RADS. This concept provides novel insight into the differential diagnosis of TCV-PTMC and C-PTMC and assists to adjust the follow-up period. While a full understanding of what causes the different subtypes of PTC may be several years away, a breakthrough leading to a meaningful relationship between its pathology and sonography could occur in the near future.

## Data availability statement

The raw data supporting the conclusions of this article will be made available by the authors, without undue reservation.

## Ethics statement

The studies involving human participants were reviewed and approved by Peking University Third Hospital Medical Science Research Ethics Committee. Written informed consent for participation was not required for this study in accordance with the national legislation and the institutional requirements.

## Author contributions

SW designed and performed research. YZ and FM performed the statistical analysis and wrote the manuscript. XH provided the materials and interpreted the data. JM collected and analyzed the data. All the authors approved the final version of the manuscript.

## Funding

This study was funded by the National Nature Science Foundation of China (No. 82072211) and Peking University Third Hospital Cohort Construction Project (No. BYSYDL2021018), which provided financial support for the whole project.

## Acknowledgments

We thank Ellen Knapp, PhD, from Liwen Bianji (Edanz) (www.liwenbianji.cn/), for editing the English text of a draft of this manuscript.

## Conflict of interest

The authors declare that the research was conducted in the absence of any commercial or financial relationships that could be construed as a potential conflict of interest.

## Publisher’s note

All claims expressed in this article are solely those of the authors and do not necessarily represent those of their affiliated organizations, or those of the publisher, the editors and the reviewers. Any product that may be evaluated in this article, or claim that may be made by its manufacturer, is not guaranteed or endorsed by the publisher.

## References

[1] Carling T Udelsman R. Thyroid cancer. Annu Rev Med (2014) 65:125–37. doi: 10.1146/annurev-med-061512-105739 24274180

[2] Fresilli D David E Pacini P Del Gaudio G Dolcetti V Lucarelli GT. Thyroid nodule characterization: How to assess the malignancy risk. update of the literature. Diagnostics (Basel) (2021) 11(8):1374. doi: 10.3390/diagnostics11081374 34441308 PMC8391491

[3] Seethala RR Baloch ZW Barletta JA Khanafshar E Mete O Sadow PM. Noninvasive follicular thyroid neoplasm with papillary-like nuclear features: A review for pathologists. Mod Pathol (2018) 31(1):39–55. doi: 10.1038/modpathol.2017.130 29052599

[4] Triggiani V Guastamacchia E Licchelli B Tafaro E. Microcalcifications and psammoma bodies in thyroid tumors. Thyroid (2008) 18(9):1017–8. doi: 10.1089/thy.2008.0082 18788924

[5] Thomas CM Asa SL Ezzat S Sawka AM Goldstein D. Diagnosis and pathologic characteristics of medullary thyroid carcinoma-review of current guidelines. Curr Oncol (2019) 26(5):338–44. doi: 10.3747/co.26.5539 PMC6821118 31708652

[6] Wang Y Li L Wang Y-XJ Feng X-L Zhao F Zou S-M. Ultrasound findings of papillary thyroid microcarcinoma: A review of 113 consecutive cases with histopathologic correlation. Ultrasound Med Biol (2012) 38(10):1681–8. doi: 10.1016/j.ultrasmedbio.2012.05.019 22920548

[7] Liu X Zhang S Gang Q Shen S Zhang J Lun Y. Interstitial fibrosis in papillary thyroid microcarcinoma and its association with biological behavior. Oncol Lett (2018) 15(4):4937–43. doi: 10.3892/ol.2018.7928 PMC5840693 29552130

[8] Sorrenti S Dolcetti V Radzina M Bellini MI Frezza F Munir K. Artificial intelligence for thyroid nodule characterization: Where are we standing? Cancers (Basel) (2022) 14(14):3357. doi: 10.3390/cancers14143357 35884418 PMC9315681

[9] Brito JP Hay ID. Management of papillary thyroid microcarcinoma. Endocrinol Metab Clin North Am (2019) 48(1):199–213. doi: 10.1016/j.ecl.2018.10.006 30717902

[10] Sugitani I Ito Y Takeuchi D Nakayama H Masaki C Shindo H. Indications and strategy for active surveillance of adult low-risk papillary thyroid microcarcinoma: Consensus statements from the Japan association of endocrine surgery task force on management for papillary thyroid microcarcinoma. Thyroid (2021) 31(2):183–92. doi: 10.1089/thy.2020.0330 PMC7891203 33023426

[11] Ulisse S Baldini E Lauro A Pironi D Tripodi D Lori E. Papillary thyroid cancer prognosis: An evolving field. Cancers (Basel) (2021) 13(21):5567. doi: 10.3390/cancers13215567 34771729 PMC8582937

[12] Morris LGT Shaha AR Tuttle RM Sikora AG Ganly I. Tall-cell variant of papillary thyroid carcinoma: A matched-pair analysis of survival. Thyroid (2010) 20(2):153–8. doi: 10.1089/thy.2009.0352 PMC3714453 20151822

[13] Poma AM Viola D Macerola E Proietti A Molinaro E De Vietro D. Tall cell percentage alone in ptc without aggressive features should not guide patients' clinical management. J Clin Endocrinol Metab (2021) 106(10):e4109–e17. doi: 10.1210/clinem/dgab388 34061965

[14] Choi YJ Shin JH J-h K SL J EJ S Oh YL. Tall cell variant of papillary thyroid carcinoma: Sonographic and clinical findings. J Ultrasound Med (2011) 30(6):853–8. doi: 10.7863/jum.2011.30.6.853 21633001

[15] Marx J. Cancer biology. all in the stroma: Cancer's cosa nostra. Science (2008) 320(5872):38–41. doi: 10.1126/science.320.5872.38 18388269

[16] Tuttle RM Haugen B Perrier ND. Updated American joint committee on Cancer/Tumor-Node-Metastasis staging system for differentiated and anaplastic thyroid cancer (Eighth edition): What changed and why? Thyroid (2017) 27(6):751–6. doi: 10.1089/thy.2017.0102 PMC5467103 28463585

[17] Tessler FN Middleton WD Grant EG. Thyroid imaging reporting and data system (Ti-rads): A user's guide. Radiology (2018) 287(1):29–36. doi: 10.1148/radiol.2017171240 29558300

[18] Ross DS Tuttle RM. Observing micopapillary thyroid cancers. Thyroid (2014) 24(1):3–6. doi: 10.1089/thy.2013.0659 24404786

[19] Wang TS Sosa JA. Thyroid surgery for differentiated thyroid cancer - recent advances and future directions. Nat Rev Endocrinol (2018) 14(11):670–83. doi: 10.1038/s41574-018-0080-7 30131586

[20] Kazaure HS Roman SA Sosa JA. Aggressive variants of papillary thyroid cancer: Incidence, characteristics and predictors of survival among 43,738 patients. Ann Surg Oncol (2012) 19(6):1874–80. doi: 10.1245/s10434-011-2129-x 22065195

[21] Wong KS Higgins SE Marqusee E Nehs MA Angell T Barletta JA. Tall cell variant of papillary thyroid carcinoma: Impact of change in who definition and molecular analysis. Endocr Pathol (2019) 30(1):43–8. doi: 10.1007/s12022-018-9561-4 30565013

[22] Vuong HG Long NP Anh NH Nghi TD Hieu MV Hung LP. Papillary thyroid carcinoma with tall cell features is as aggressive as tall cell variant: A meta-analysis. Endocr Connect (2018) 7(12):R286–R93. doi: 10.1530/EC-18-0333 PMC6240142 30352403

[23] Kant R Davis A Verma V. Thyroid nodules: Advances in evaluation and management. Am Fam Physician (2020) 102(5):298–304. 32866364

[24] Baek HJ Kim DW Shin GW Heo YJ Baek JW Lee YJ. Ultrasonographic features of papillary thyroid carcinomas according to their subtypes. Front Endocrinol (Lausanne) (2018) 9:223. doi: 10.3389/fendo.2018.00223 29867759 PMC5951938

[25] Hernandez-Prera JC Machado RA Asa SL Baloch Z Faquin WC Ghossein R. Pathologic reporting of tall-cell variant of papillary thyroid cancer: Have we reached a consensus? Thyroid (2017) 27(12):1498–504. doi: 10.1089/thy.2017.0280 29020884

[26] Tappouni RR Itri JN McQueen TS Lalwani N Ou JJ. Acr Ti-rads: Pitfalls, solutions, and future directions. Radiographics (2019) 39(7):2040–52. doi: 10.1148/rg.2019190026 31603734

[27] Zhang J-Z Hu B. Sonographic features of thyroid follicular carcinoma in comparison with thyroid follicular adenoma. J Ultrasound Med (2014) 33(2):221–7. doi: 10.7863/ultra.33.2.221 24449724

[28] Das DK. Functional state of cells during their life and on their journey toward inactivity and death: Search for morphological evidence in thyroid fine needle aspiration smears. J Cytol (2018) 35(3):131–8. doi: 10.4103/JOC.JOC_43_18 PMC6060585 30089940

[29] Das DK Sheikh ZA George SS Al-Baquer T Francis IM. Papillary thyroid carcinoma: Evidence for intracytoplasmic formation of precursor substance for calcification and its release from well-preserved neoplastic cells. Diagn Cytopathol (2008) 36(11):809–12. doi: 10.1002/dc.20898 18831027

[30] Gubbiotti MA Livolsi V Montone K Baloch Z. Papillary thyroid microcarcinomas: Does subtyping predict aggressive clinical behavior? Hum Pathol (2021) 114:28–35. doi: 10.1016/j.humpath.2021.04.015 33971214

[31] Gallagher D Heymsfield SB Heo M Jebb SA Murgatroyd PR Sakamoto Y. Healthy percentage body fat ranges: An approach for developing guidelines based on body mass index. Am J Clin Nutr (2000) 72(3):694–701. doi: 10.1093/ajcn/72.3.694 10966886

[32] Avgerinos KI Spyrou N Mantzoros CS Dalamaga M. Obesity and cancer risk: Emerging biological mechanisms and perspectives. Metabolism (2019) 92:121–35. doi: 10.1016/j.metabol.2018.11.001 30445141

